# Excessive Astrocyte-Derived Neurotrophin-3 Contributes to the Abnormal Neuronal Dendritic Development in a Mouse Model of Fragile X Syndrome

**DOI:** 10.1371/journal.pgen.1003172

**Published:** 2012-12-27

**Authors:** Qi Yang, Bin Feng, Kun Zhang, Yan-yan Guo, Shui-bing Liu, Yu-mei Wu, Xiao-qiang Li, Ming-gao Zhao

**Affiliations:** Department of Pharmacology, School of Pharmacy, Fourth Military Medical University, Xi'an, China; University of British Columbia, Canada

## Abstract

Fragile X syndrome (FXS) is a form of inherited mental retardation in humans that results from expansion of a CGG repeat in the *Fmr1* gene. Recent studies suggest a role of astrocytes in neuronal development. However, the mechanisms involved in the regulation process of astrocytes from FXS remain unclear. In this study, we found that astrocytes derived from a Fragile X model, the *Fmr1* knockout (KO) mouse which lacks FMRP expression, inhibited the proper elaboration of dendritic processes of neurons *in vitro*. Furthermore, astrocytic conditioned medium (ACM) from KO astrocytes inhibited proper dendritic growth of both wild-type (WT) and KO neurons. Inducing expression of FMRP by transfection of FMRP vectors in KO astrocytes restored dendritic morphology and levels of synaptic proteins. Further experiments revealed elevated levels of the neurotrophin-3 (NT-3) in KO ACM and the prefrontal cortex of *Fmr1* KO mice. However, the levels of nerve growth factor (NGF), brain-derived neurotrophic factor (BDNF), glial cell-derived neurotrophic factor (GDNF), and ciliary neurotrophic factor (CNTF) were normal. FMRP has multiple RNA–binding motifs and is involved in translational regulation. RNA–binding protein immunoprecipitation (RIP) showed the *NT-3* mRNA interacted with FMRP in WT astrocytes. Addition of high concentrations of exogenous NT-3 to culture medium reduced the dendrites of neurons and synaptic protein levels, whereas these measures were ameliorated by neutralizing antibody to NT-3 or knockdown of NT-3 expression in KO astrocytes through short hairpin RNAs (shRNAs). Prefrontal cortex microinjection of WT astrocytes or NT-3 shRNA infected KO astrocytes rescued the deficit of trace fear memory in KO mice, concomitantly decreased the NT-3 levels in the prefrontal cortex. This study indicates that excessive NT-3 from astrocytes contributes to the abnormal neuronal dendritic development and that astrocytes could be a potential therapeutic target for FXS.

## Author Summary

Fragile X syndrome is a form of inherited mental retardation in humans that results from expansion of a CGG repeat in the *Fmr1* gene. Recent studies suggest that astrocytes play a role in neuronal growth. In this study, we find that astrocytes derived from a Fragile X model, the *Fmr1* knockout (KO) mouse, inhibit the proper elaboration of dendritic processes of neurons *in vitro*. Excessive neurotrophin-3 (NT-3) is released in the astrocytes from *Fmr1* KO mice. Blockage of NT-3 by neutralizing antibodies and knockdown of NT-3 by using short hairpin RNAs (shRNAs) in *Fmr1* KO astrocytes can rescue the neuronal dendritic development. *In vivo* experiments show that prefrontal cortex microinjection of WT astrocytes or NT-3 shRNA–infected KO astrocytes rescues the deficit of trace fear memory in KO mice. This study provides the evidence that a lack of FMRP leads to an overexpression of NT-3, which reduces dendritic growth in neurons.

## Introduction

Fragile X syndrome is a form of inherited mental retardation in humans that results from expansion of a CGG repeat in the *Fmr1* gene on the X chromosome [1], [2]. This syndrome is characterized by low intelligence quotient, attention deficits, and anxiety [3]–[6]. As an mRNA binding protein, FMRP is associated with polyribosomes and involved in the translational efficiency and/or trafficking of certain mRNAs [7]. FMRP is widely expressed in the brain [8], [9], and its absence is expected to disrupt the synthesis and/or the subcellular localization of several proteins, which is important in long-term synaptic plasticity [10], [11]. *Fmr1* knockout (KO) mice serve as a model to study fragile X mental retardation [12], [13]. Our previous study has identified FMRP as a key messenger for dopamine modulation in the forebrain and provided insights on the cellular and molecular mechanisms underlying FXS [14]. However, the cellular pathophysiology of FXS is still under discussion.

Emerging evidence suggests that glia may also be involved in the development of FXS. For example, astrocytes, the major glia of the central nervous system (CNS), have been shown to regulate the stability, dynamics, and maturation of dendritic spines [15], [16] and take part in the regulation of synaptic plasticity and synaptic transmission [17], [18]. FMRP is expressed in the astrocyte lineage during development [19], and a lack of FMRP in astrocytes affects the dendritic morphology of neurons [20]. Evidence is steadily implicating astrocytes in synaptic maturation and elimination, suggesting that FMRP may be essential to the role of astrocytes in synaptogenesis during development [19]. However, the underlying mechanisms of astrocytes in regulating neuronal dendritic development in FXS are still unclear.

In this study, we found that a lack of FMRP leads to an overexpression of neurotrophin-3 (NT-3) and this in turn reduces dendritic growth in neurons. Therefore, excessive NT3 from astrocytes contributed to the dendritic developmental disorder of *Fmr1* KO mice. The present study indicates an important role of normal astrocyte secretion in neuronal dendritic development and provides a potential target for FXS treatment.

## Results

### Astrocytes affect the neuronal dendritic development in *Fmr1* KO mice

**Figure 1:**
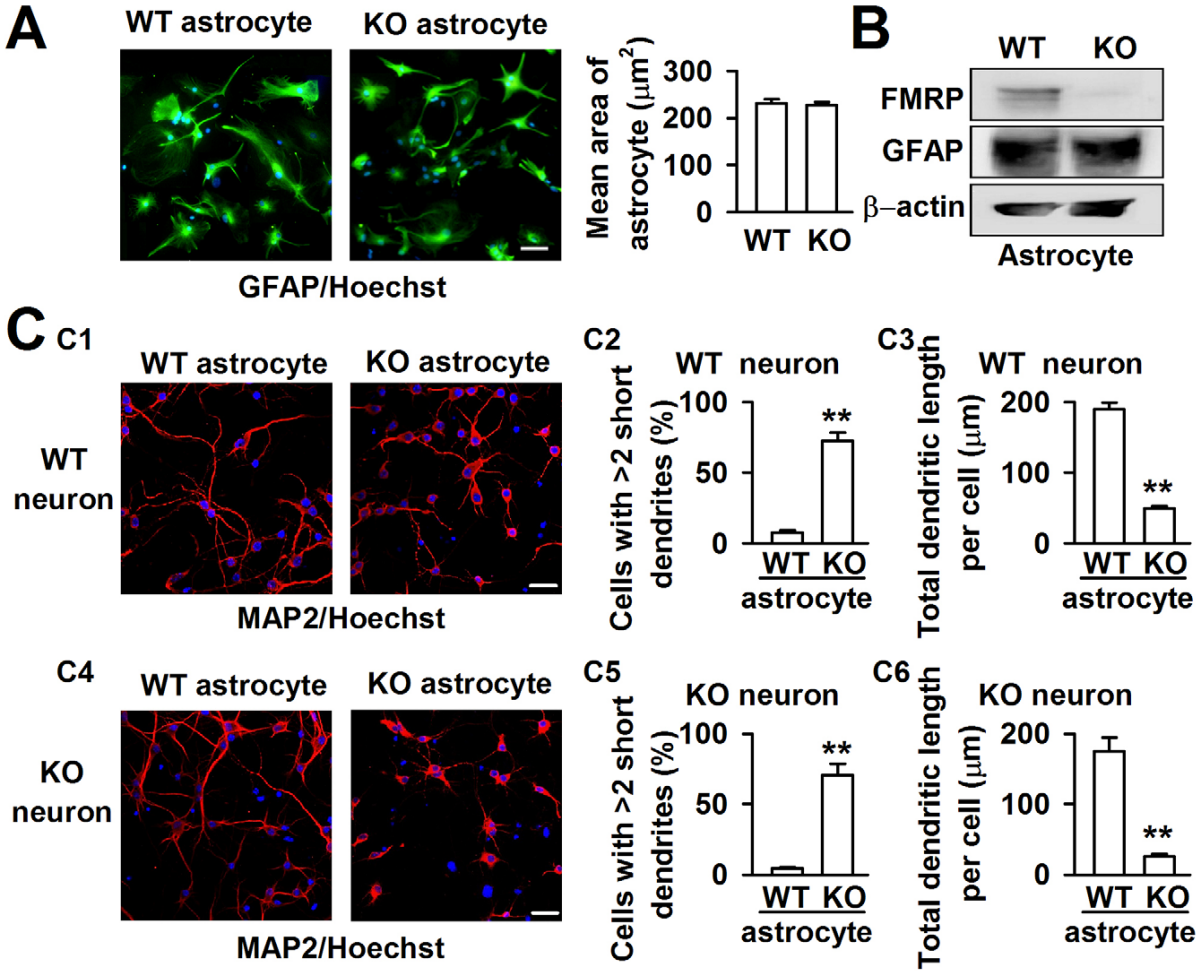
Different dendritic morphology of cortical neurons cocultured with WT and KO astrocytes. A, Astrocytes from WT and KO mice indicated by the astrocytic marker GFAP (green) and nucleic staining Hoechst33258 (blue). Scale bar = 50 µm. The average area of single astrocyte was similar between the WT and KO mice. *n = *136 WT astrocytes, *n = *145 KO astrocytes from three independent experiments. B, Western blot analysis showed the expression of FMRP in WT astrocytes, but not in KO astrocytes. C, Neurons stained with the neuronal dendritic marker MAP2 (red) and nucleic staining Hoechst33258 (blue) at DIV 7. C1, The images are representative WT neurons cocultured with WT and KO astrocytes, respectively. Scale bar = 50 µm. C2, Percentage of WT neurons with at least two short (<50 µm) dendrites. C3, The total dendritic length per cell of WT neurons cocultured with WT and KO astrocytes. C2–C3: the number of neurons in WT astrocytes group: n* = *235 neurons, KO astrocytes group: *n = *212 neurons. Data were from three independent experiments. **P<0.01 compared with the WT astrocytes. C4, The images are representative KO neurons cocultured with WT and KO astrocytes, respectively. Scale bar = 50 µm. C5, Percentage of KO neurons with at least two short (<50 µm) dendrites. C6, The total dendritic length per cell of KO neurons cocultured with WT and KO astrocytes. C5–C6: the number of neurons in WT astrocytes group: *n = *249 neurons, KO astrocytes group: *n = *198 neurons. Data were from three independent experiments. **P<0.01 compared with the WT astrocytes.

FXS patients and *Fmr1* KO mice have been known to have abnormal dendritic arbors with increased branch density in neurons [21]. Astrocytes are required for the efficient formation, maturation, and maintenance of synapses [17], [18]. To explore the role of astrocytes in FXS, we isolated astrocytes from both WT and KO newborn mice with over 95% purity, as determined by the cell specific marker GFAP staining. The average area of single astrocytes was not different between the WT and KO astrocytes (Figure 1A). Western blot analysis indicated that the astrocytes expressed FMRP in WT mice but not in *Fmr1* KO mice (Figure 1B). This result is consistent with that of previous research [19]. To investigate the role of astrocytes in neuronal development, a coculture system of astrocytes and neurons was adopted to mimic the situation of astrocytes and neurons in the brain. The neuronal dendrites were stained with the neuronal marker microtubule-associated protein 2 (MAP2). Based on our observations, the dendritic morphology was quite different in WT cortical neurons when cocultured with WT and KO astrocytes (Figure 1C1). We considered neurons with more than two short (<50 µm) dendrites as abnormal dendritic morphological neurons [22]. WT neurons cocultured with WT astrocytes exhibited only 7.6±1.9% cells with >2 short dendrites, whereas 72.2±6.1% cells with >2 short dendrites were observed when the WT neurons were cocultured with KO astrocytes (Figure 1C2). To evaluate more precisely, we performed a detailed morphological analysis, and found that the total dendritic length per cell was decreased by 74.2±5.8% when we compared neurons cocultured with KO astrocytes to those with WT astrocytes (Figure 1C3). A similar phenomenon was observed in KO neurons cocultured with WT and KO astrocytes (Figure 1C4). The cells with >2 short dendrites were 4.5±0.8% and 70.5±8.1% when cocultured with WT and KO astrocytes, respectively (Figure 1C5). The total dendritic length per cell of KO neurons was decreased by 85.3±6.4% when compared neurons cocultured with KO astrocytes to those with WT astrocytes (Figure 1C6). These results indicate that KO astrocytes alter the dendritic morphology of WT neurons, and WT astrocytes can prevent the abnormal morphology of KO neurons. Thus, astrocytes may play an important role in the development of FXS.

### Factors released from *Fmr1* KO astrocytes cause abnormal neuronal growth

**Figure 2:**
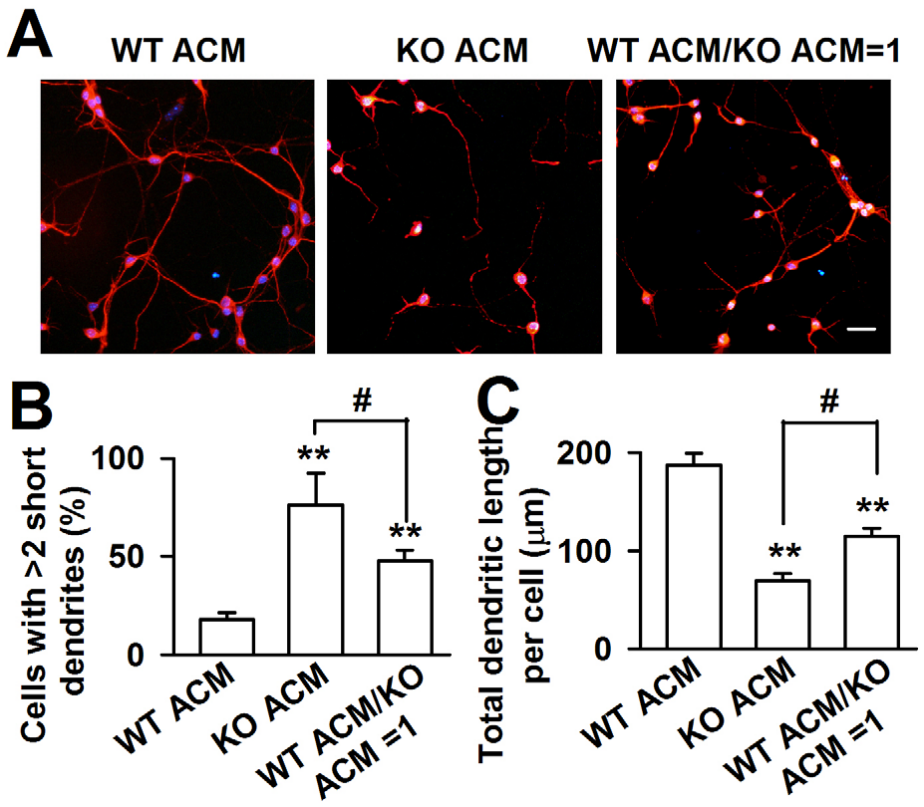
*Fmr1* KO ACM altered the morphology of WT cortical neurons. A, WT neurons were cultured in ACM from WT or KO astrocytes, as well as the mixture ACM from WT and KO astrocytes at a ratio of 1∶1 for seven days. Scale bar = 50 µm. B, Percentage of neurons with at least two short (<50 µm) dendrites. C, The total dendritic length per cell of neurons cultured with WT ACM, KO ACM, and WT/KO ACM. B–C: the number of neurons in WT ACM: *n = *251 neurons, KO ACM: *n = *68 neurons, WT/KO ACM: *n = *192 neurons. Data were from three independent experiments. **P<0.01 compared with the WT ACM; ^#^ *P*<0.05 compared with the KO ACM.

To confirm that KO astrocytes resulted in abnormal dendritic morphology via the secretion of soluble factors, astrocytic conditioned medium (ACM) was generated to culture the neurons. The WT cortical neurons were plated in a low-density culture with WT and KO ACM respectively. Within 24 h, the neuronal survival was indistinguishable (data not shown). At DIV 7, we found a great variance of neuronal dendritic morphology between WT and KO ACM-treated neurons. The dendrites of the neurons treated with KO ACM were shorter and smaller; however, the neuronal densities were not different (Figure 2A). Compared with the WT ACM-treated neurons, the number of cells with more than two short dendrites in the KO ACM-treated neurons increased by 58.1±19.8% (Figure 2B), the total dendritic length per cell decreased by 62.9±9.5% (Figure 2C). The 1∶1 ratio of WT and KO ACM showed a much better dendritic morphology and length than that in only KO ACM (Figure 2B and 2C), suggesting that WT astrocytes can alleviate the neuronal abnormality caused by KO astrocytes.

### FMRP in astrocytes is crucial for neuronal dendritic development

**Figure 3:**
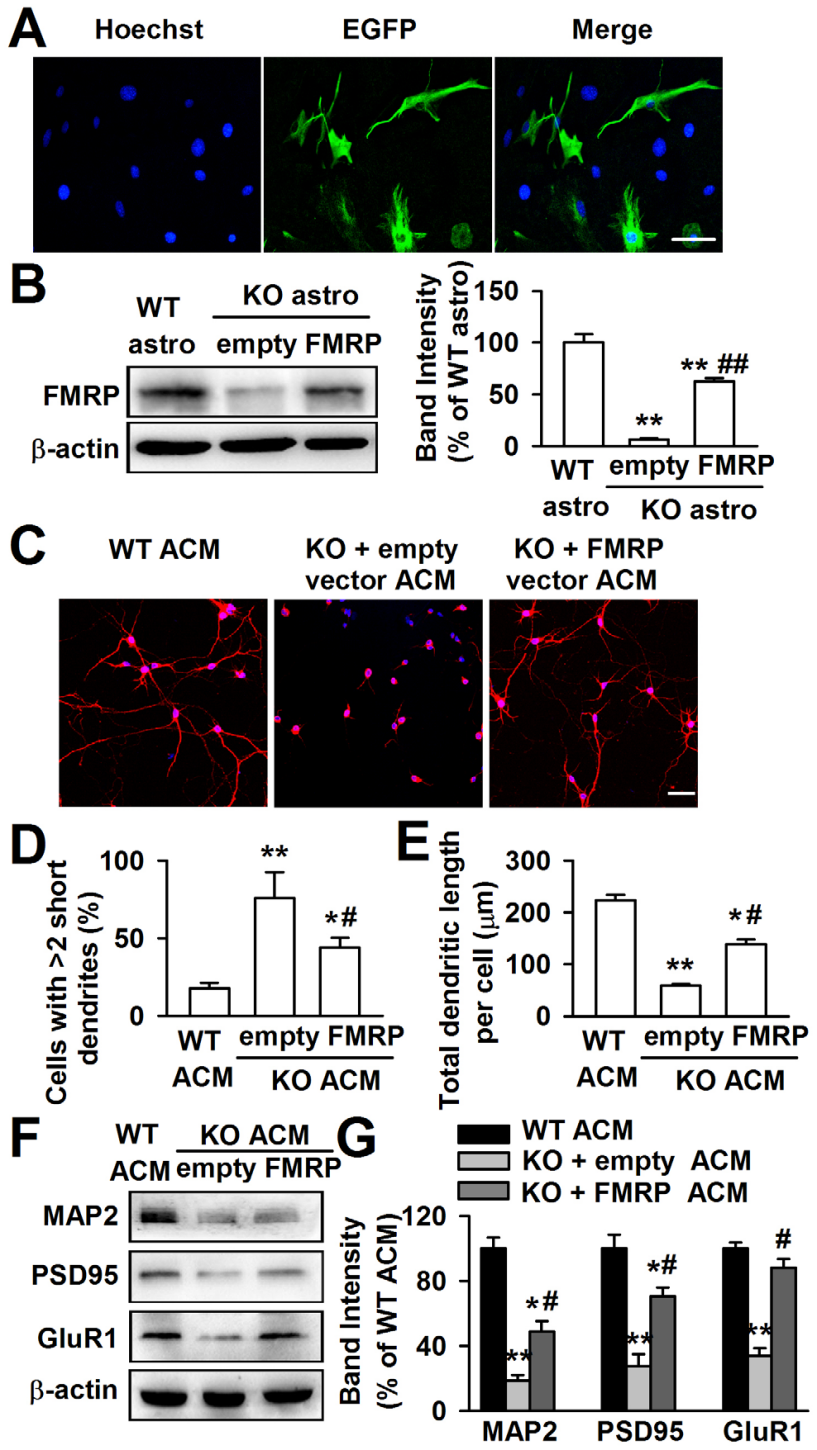
Expression of FMRP reversed neuronal growth in *Fmr1* KO ACM. Cultured astrocytes at DIV 7 were transfected with FMRP expression vectors for 48 h. A, Confocal images showing the transfection efficiency of FMRP vectors. Nuclei were stained with Hoechst33258 (blue). EGFP-positive astrocytes (green) indicated the successful transfection of FMRP vectors. Scale bar = 50 µm. B, Transfection of FMRP vectors resulted in the expression of FMRP in KO astrocytes, whereas the negative empty vectors did not. *n = *6 wells from three independent experiments. **P<0.01 compared with the WT astrocytes; ^##^ *P*<0.01 compared with the empty control group. C, Neuronal dendritic development was significantly improved in ACM of FMRP vectors transfected KO astrocytes. Scale bar = 50 µm. D, Quantification of neurons with at least two short (<50 µm) dendrites. E, Quantification of the total dendritic length per cell. D–E: the number of neurons in WT ACM: *n = *213 neurons, KO + empty ACM: *n = *236 neurons, KO + FMRP ACM: *n = *185 neurons. Data were from three independent experiments. *P<0.05, **P<0.01 compared with the WT ACM; ^#^ *P*<0.05 compared with the empty vector transfected KO ACM. F, The expressions of MAP2, PSD95, and GluR1 were detected by Western blot. G, Band intensities were quantified as percentage of values from WT ACM-treated neurons. n = 6 wells from three independent experiments. *P<0.05, **P<0.01 compared with the WT ACM; ^#^ *P*<0.05 compared with the empty vector transfected KO ACM.

If the aberrant neuronal dendritic morphology is due to the lack of FMRP, the expression of FMRP in *Fmr1* KO astrocytes is expected to rescue this deficit. The *Fmr1* KO astrocytes expressed the enhanced green fluorescent protein (EGFP) when they were transfected successfully with FMRP constructs. As shown in the Figure 3A, the EGFP positive astrocytes were 52.3%±5.6% after 48 h of transfection. The transfection of FMRP vectors into KO astrocytes resulted in a 62.5%±7.2% FMRP protein expression of WT astrocytes (Figure 3B). The ACM of FMRP-transfected KO astrocytes markedly altered the neuronal dendritic morphology as compared to the empty vector transfected KO ACM (Figure 3C). The number of abnormal neurons was decreased by 42.1%±22.4% (Figure 3D), and the total dendritic length per cell was increased by 139.7%±16.9% (Figure 3E). In addition, the ACM of FMRP-transfected KO astrocytes also reversed the decreased levels of MAP2, postsynaptic protein PSD95, and glutamate receptor subunit GluR1 in the cultured neurons as compared to the empty vector transfected KO ACM-cultured neurons (Figure 3F and 3G). Taken together, these results indicate that FMRP expression in astrocytes is important for neuronal development.

### High levels of NT-3 are released from *Fmr1* KO astrocytes

**Figure 4:**
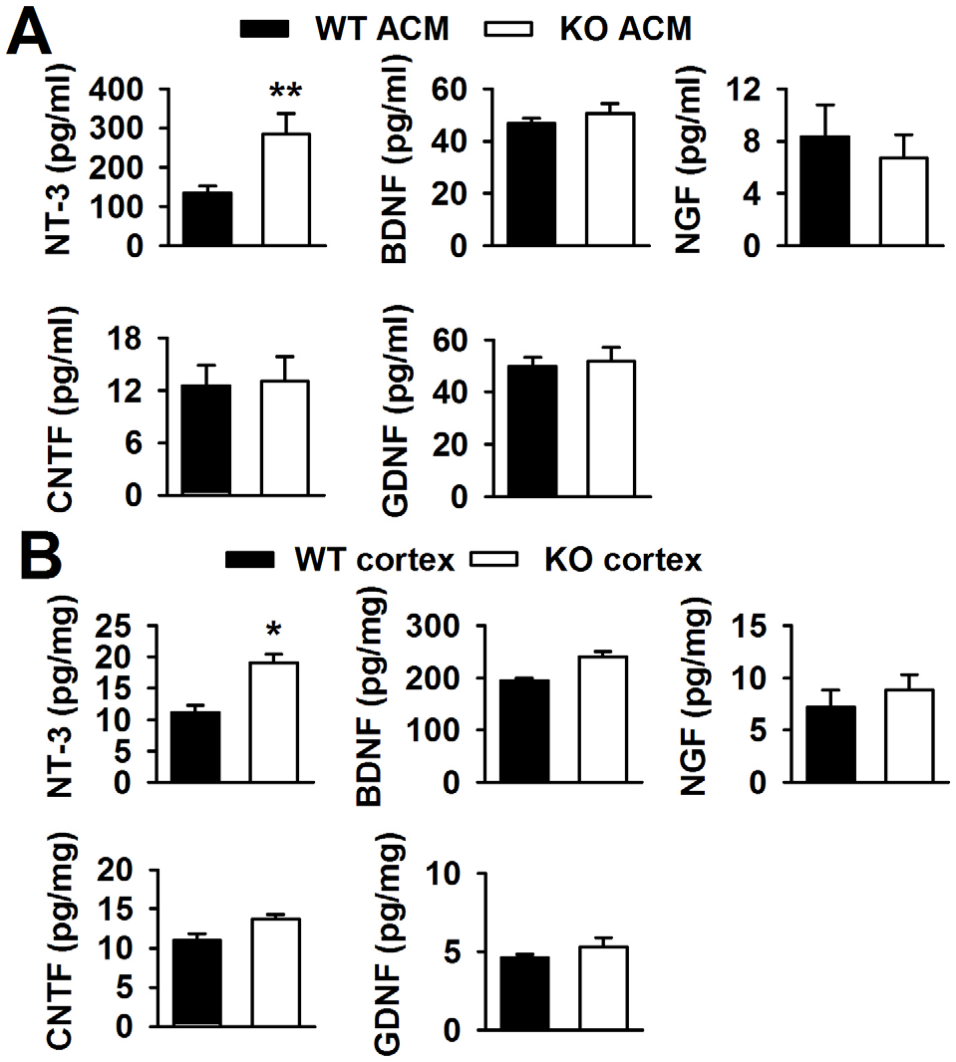
Levels of neurotrophic factors *in vitro* and *in vivo*. A, NT-3 levels in KO ACM were more than twofold of those in WT ACM. Levels of BDNF, NGF, CNTF, and GDNF were similar between the WT and KO ACM. *n = *6 wells from three independent experiments. **P<0.01 compared with the WT ACM. B, NT-3 levels were higher in KO cerebral cortex than those in WT cerebral cortex from mice 3∼4 weeks old. The levels of other four factors had no difference between WT and KO cerebral cortex. *n = *6 mice in each group. *P<0.05 compared with the WT cerebral cortex.

Neurotrophic factors in the brain can promote neuronal survival and differentiation during development and participation in plasticity-related processes, such as NGF [23], NT-3 [24], and BDNF [25]. Thus, we detected some neurotrophic factors in the ACMs using ELISA kits. The ELISA kits used in the present study were designed for the total target proteins, not uniformly specific for mature and immature forms of the proteins. Interestingly, we found that BDNF, NGF, CNTF, and GDNF were almost unchanged in KO ACM compared with WT ACM. However, the NT-3 concentration in KO ACM (284.3±54.3 pg/ml) was more than two folds of that in WT ACM (133.4±18.4 pg/ml, Figure 4A). At aged 3∼4 weeks old mice, the NT-3 levels in the cerebral cortex of *Fmr1* KO mice were also higher (19.1±1.4 pg/mg protein) than that of WT mice (11.1±1.2 pg/mg protein, Figure 4B). The NT-3 levels in older mice were not studied, because in adult tissues, neither WT nor KO astrocytes would express FMRP [19], [26]. Unlike NT-3, the other four neurotrophic factors remained unchanged both *in vitro* and *in vivo* (Figure 4A and 4B). Additionally, the levels of NT-3, BDNF, NGF, CNTF, and GDNF from the neuron culture medium were too low to detect. Therefore, excessive NT-3 released from the astrocytes of KO mice might be one of the causes for abnormal neuronal growth.

### FMRP interacts directly with *NT-3* mRNA

**Figure 5:**
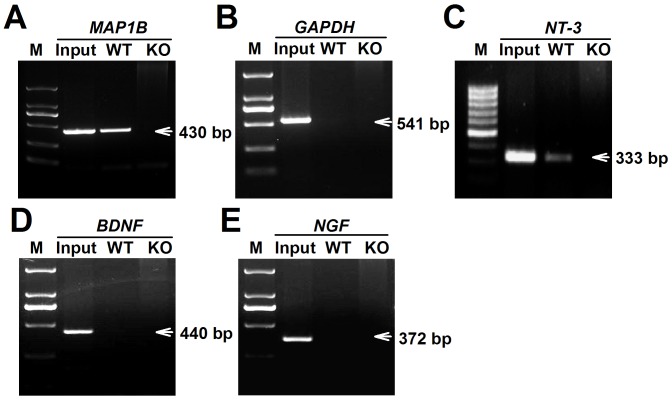
FMRP interacted directly with *NT-3* mRNA. WT and KO astrocytic lysates were immunoprecipitated with FMRP antibody. RT-PCR was performed using specific primers for the *MAP1B*, *GAPDH*, *NT-3*, *BDNF*, and *NGF* mRNAs. Input was used as 5% of WT astrocytic lysates. A, A known FMRP-interacting mRNA, *MAP1B* was co-precipitated as the positive control. B, *GAPDH* mRNA was as a negative control. C, *NT-3* mRNA was found in FMRP immunoprecipitates from WT astrocytes but not from KO astrocytes. D and E, The homology classes of *BDNF* and *NGF* mRNAs were not found binding with FMRP. Data were from four independent experiments.

To determine the reason for high levels of NT-3 released from *Fmr1* KO astrocytes, the *NT-3* mRNA levels of WT and KO astrocytes were examined. We found that the *NT-3* mRNA levels in WT and KO astrocytes did not change (Figure S1A). However, the NT-3 protein levels were higher in KO astrocytes than in WT astrocytes (Figure S1B and S1C). These data suggest that the aberrant secretion of NT-3 in KO astrocytes is not caused by transcriptional regulation, but likely by protein synthesis. As we know, FMRP has multiple RNA-binding motifs and is involved in translational regulation [7]. To verify that *NT-3* mRNA is a potential target of FMRP, we performed RIP to examine whether *NT-3* mRNA directly binds with FMRP. We found the *NT-3* mRNA in FMRP immunoprecipitates from WT astrocytes but not from *Fmr1* KO astrocytes (Figure 5C). A known FMRP-interacting mRNA, *MAP1B* [27]–[29], was co-precipitated as the positive control (Figure 5A), whereas a negative control mRNA (*GAPDH*) was not co-precipitated with the FMRP (Figure 5B). The homology classes of *BDNF* and *NGF* mRNA did not bind with FMRP (Figure 5D and 5E). Our findings indicate that *NT-3* mRNA is part of the FMRP-mRNA complex and the translation of *NT-3* mRNA is regulated by FMRP.

### NT-3 is responsible for the abnormal neuronal growth caused by KO ACM

**Figure 6:**
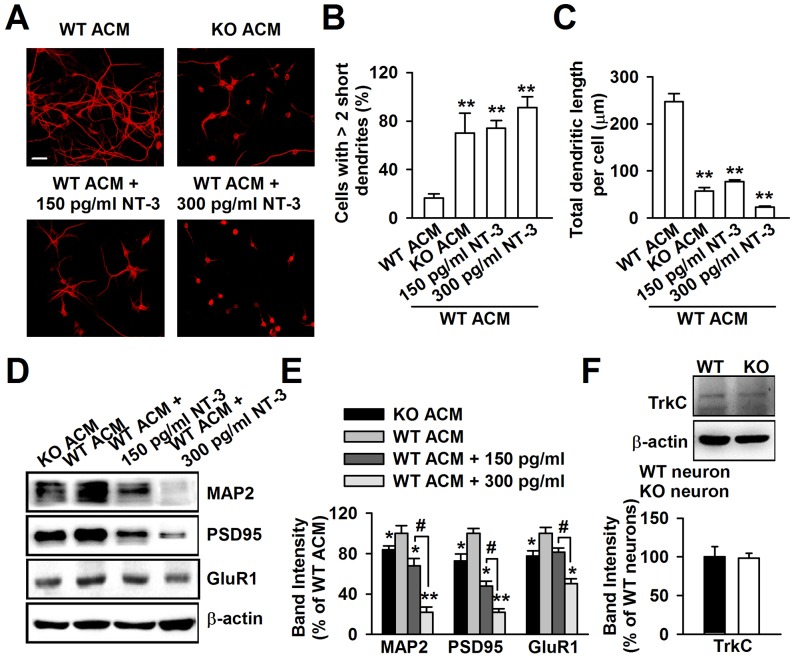
Excessive NT-3 was neurotoxic to neuronal development. A, High levels of exogenous NT-3 caused neuronal dendritic developmental disorder. Scale bar = 50 µm. B, Quantification of neurons with at least two short (<50 µm) dendrites. C, Quantification of the total dendritic length per cell. B–C: the number of neurons in WT ACM: *n = *242 neurons, KO ACM: *n = *215 neurons, WT ACM +150 pg/ml NT-3: *n = *168 neurons, WT ACM +300 pg/ml NT-3: *n = *132 neurons. Data were from three independent experiments. **P<0.01 compared with the WT ACM. D, Western blot analysis of MAP2, PSD95, and GluR1 in WT neurons after NT-3 treatment. E, Band intensities were quantified as percentage of values from WT ACM-treated neurons. *n* = 6 wells from three independent experiments. *P<0.05, **P<0.01 compared with WT ACM-treated group; ^#^ *P*<0.05 compared with WT ACM +150 pg/ml exogenous NT-3 groups. F, The expression of TrkC receptor in WT and KO neurons were detected by Western blot. *n = *6 wells from three independent experiments.

To determine whether NT-3 is responsible for the dendritic disorder caused by KO ACM, we added exogenous NT-3 into WT ACM to observe the change in neuronal morphology. According to the difference between the NT-3 levels in WT and KO ACM, we set a low (150 pg/ml) and a high (300 pg/ml) dose of NT-3 in WT ACM. The neurons were stained with the dendritic protein MAP2 (Figure 6A). We observed that the dendrites of the neurons treated with 150 pg/ml of NT-3 had a similar morphology to those of KO ACM-treated neurons, as shown by the smaller and shorter dendrites (Figure 6B and 6C). Furthermore, the neurons treated with 300 pg/ml of NT-3 exhibited a worse developmental morphology (Figure 6B and 6C). In contrast to NT-3, the other two neurotrophins, NGF and BDNF, had no effects on neuronal development (Figure S2A–S2C). The dendritic damage was validated further by the detection of the postsynaptic elements of excitatory synapses (Figure 6D). The expressions of MAP2, PSD95, and GluR1 were significantly decreased in neurons treated with NT-3 as compared to the WT ACM-treated neurons. Moreover, this reduction was NT-3 dose-dependent (Figure 6E). Because the major actions of NT-3 on neurons are produced by binding to the high affinity receptor tyrosine kinase C (TrkC), we further studied the TrkC expression in neurons, and found that WT and KO neurons expressed similar levels of the TrkC (Figure 6F).

### Neutralization of NT-3 in KO ACM prevents abnormal neuronal growth

**Figure 7:**
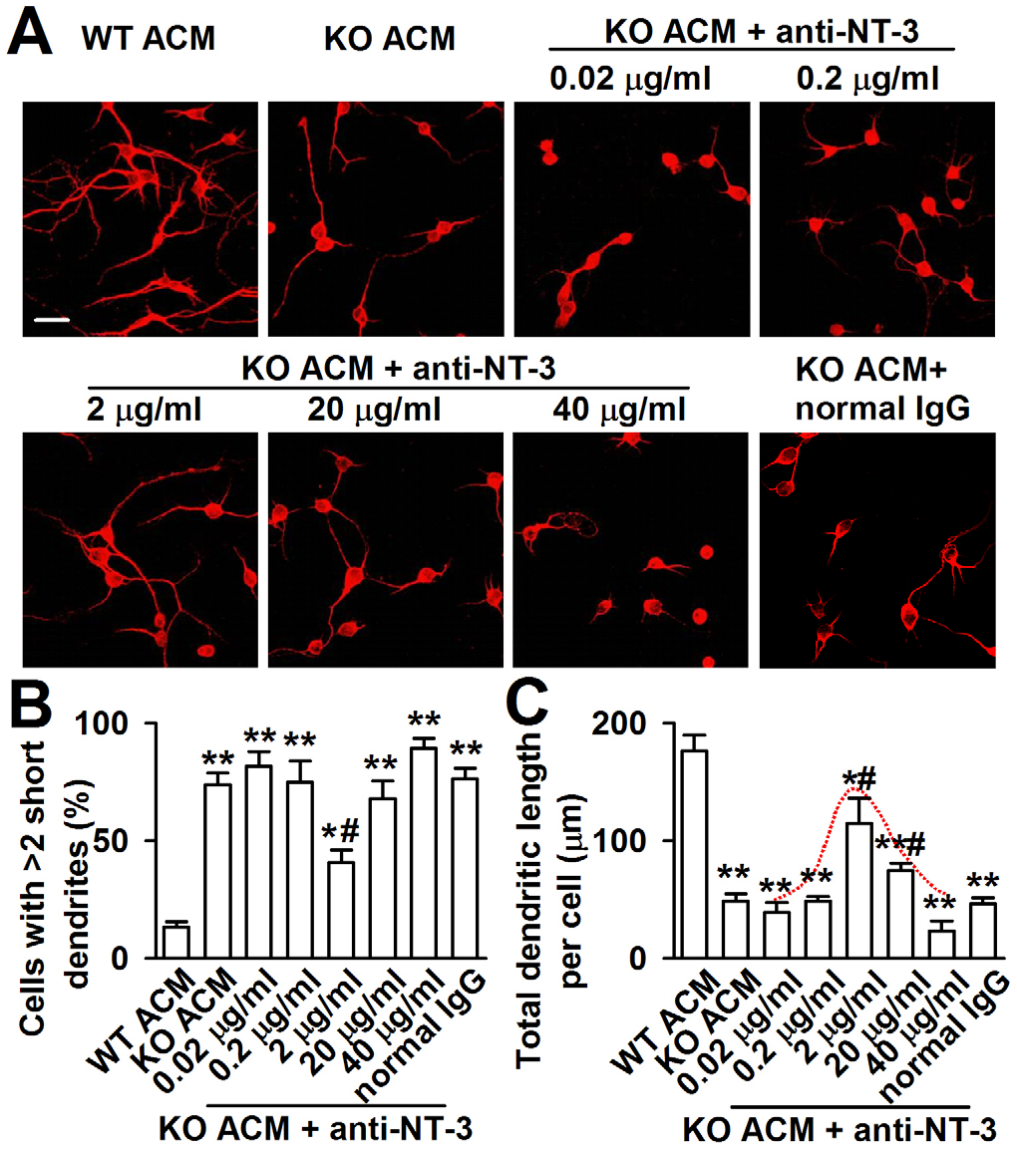
Effects of NT-3 neutralizing antibody on the neuronal dendritic growth. A, Representative images of each treatment are shown. The final concentration of NT-3 neutralizing antibody in cultured KO ACM of antibody was 0.02, 0.2, 2, 20 and 40 µg/ml, respectively. A normal rabbit IgG served as the negative control. Scale bar = 50 µm. B, Quantification of neurons with at least two short (<50 µm) dendrites. C, Quantification of the total dendritic length per cell. B–C: the number of neurons in WT ACM: *n = *186 neurons, KO ACM: *n = *192 neurons, KO ACM +0.02 µg/ml antibody: *n = *184 neurons, KO ACM +0.2 µg/ml antibody: *n = *169 neurons, KO ACM +2 µg/ml antibody: *n = *236 neurons, KO ACM +20 µg/ml antibody: *n = *249 neurons, KO ACM +40 µg/ml antibody: *n = *194 neurons. Data were from three independent experiments. *P<0.05, **P<0.01 compared with the WT ACM; ^#^ *P*<0.05 compared with the KO ACM.

To provide direct evidence that excessive astrocyte-derived NT-3 inhibits dendritic development, we analyzed the effects of neutralizing antibody against NT-3 on neuronal dendritic development after KO ACM treatment. We found that the effect of NT-3 neutralization on neuronal dendritic development was dose dependent (Figure 7A–7C). The classic bell-shaped curve depicted dendritic growth in response to NT-3 antibody (Figure 7C). With the increase of dose, the neutralizing antibody to NT-3 gradually rescued dendritic growth, and 2 µg/ml of NT-3 antibody took the best effects to rescue the dendritic growth. However, high doses of NT-3 antibody (20 and 40 µg/ml) exhibited less effect to the dendritic growth (Figure 7B and 7C). This suggests that excessive NT-3 contributes to abnormal dendritic morphology while physiological levels of NT-3 are also necessary for dendritic development.

### Down-regulation of NT-3 expression from KO astrocytes rescues neuronal growth

**Figure 8:**
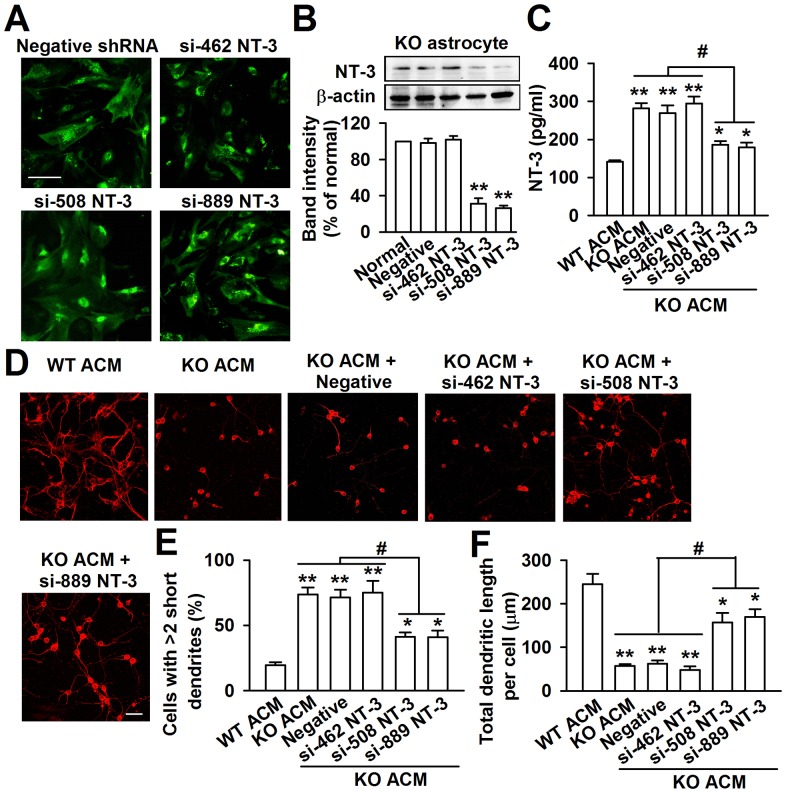
Knockdown of NT-3 in *Fmr1* KO astrocytes rescued aberrant neuronal morphology. A, GFP-positive astrocytes indicated the successful infection of NT-3 shRNAs. Scale bar = 50 µm. B, NT-3 expression in KO astrocytes was detected by Western blot 24 h after lentivirus infection. NT-3 expression was successfully inhibited by si-508 and si-889. *n = *6 wells from three independent experiments. **P<0.01 compared with the negative shRNA-treated group. C, Infection with NT-3 shRNAs si-508 and si-889 resulted in the decrease of NT-3 levels in KO ACM. The shRNA si-462 and negative control did not change the levels of NT-3. *n = *6 wells from three independent experiments. *P<0.05, **P<0.01 compared with the WT ACM; ^#^ *P*<0.05 compared with the KO ACM, KO ACM + negative and si-462 shRNAs. D, Representative immunofluorescent images showed the abnormal neuronal dendritic morphology in KO ACM, and knockdown of NT-3 by si-508 and si-889 reversed the abnormal neuronal dendritic morphology. Scale bar = 50 µm. E, Quantification of neurons with at least two short (<50 µm) dendrites. F, Quantification of the total dendritic length per cell. E–F: the number of neurons in WT ACM: *n = *267 neurons, KO ACM: *n = *185 neurons, KO ACM + negative shRNA: *n = *162 neurons, KO ACM + si-462 shRNA: *n = *176 neurons, KO ACM + si-508 shRNA: *n = *213 neurons, KO ACM + si-889 shRNA: *n = *236 neurons. Data were from three independent experiments. *P<0.05, **P<0.01 compared with the WT ACM; ^#^ *P*<0.05 compared with the KO ACM, KO ACM + negative and si-462 shRNAs.

To further evaluate the biological role of astrocyte-derived NT-3 in the dendritic disorder, we knocked down the NT-3 via shRNA infection. The KO astrocytes expressed the green fluorescent protein (GFP) when they were infected successfully with NT-3 shRNA. As shown in the Figure 8A, the GFP positive astrocytes were 92.5%±6.6% after 24 h of shRNA infection (Figure 8A). This approach successfully reduced the NT-3 level by two shRNAs for NT-3, while shRNA si-462 was incompetent to knockdown NT-3 expression (Figure 8B). The NT-3 protein band intensity reduced to 31.6%±6.2% and 26.5%±3.6% of control by shRNAs si-508 and si-889 respectively (Figure 8B). Consistently, the levels of the NT-3 decreased significantly to 186.0±9.5 pg/ml and 179.2±12.4 pg/ml in the ACM of KO astrocytes by shRNAs si-508 and si-889 respectively (Figure 8C). As expected, the inhibition of KO astrocyte-derived NT-3 partially rescued the aberrant neuronal dendritic morphology (Figure 8D). Compared to KO ACM treatment, the number of abnormal neurons was decreased by shRNAs si-508 and si-889, but not by shRNA si-462 and the negative control (Figure 8E). Consistently, the total dendritic length per cell was increased by shRNAs si-508 and si-889, but not by shRNA si-462 and negative control (Figure 8F). Next, the effect of NT-3 knockdown on the synaptic protein levels was observed. As shown in Figure S3, the levels of MAP2, PSD95, and GluR1 were partially rescued in NT-3 shRNA-infected KO ACM-cultured neurons as compared to those in KO ACM-cultured neurons. These data indicate that interfering NT-3 expression in KO astrocytes can prevent abnormal neuronal growth in KO ACM.

### Normal astrocytes *in vivo* rescue the trace fear memory of *Fmr1* KO mice

**Figure 9:**
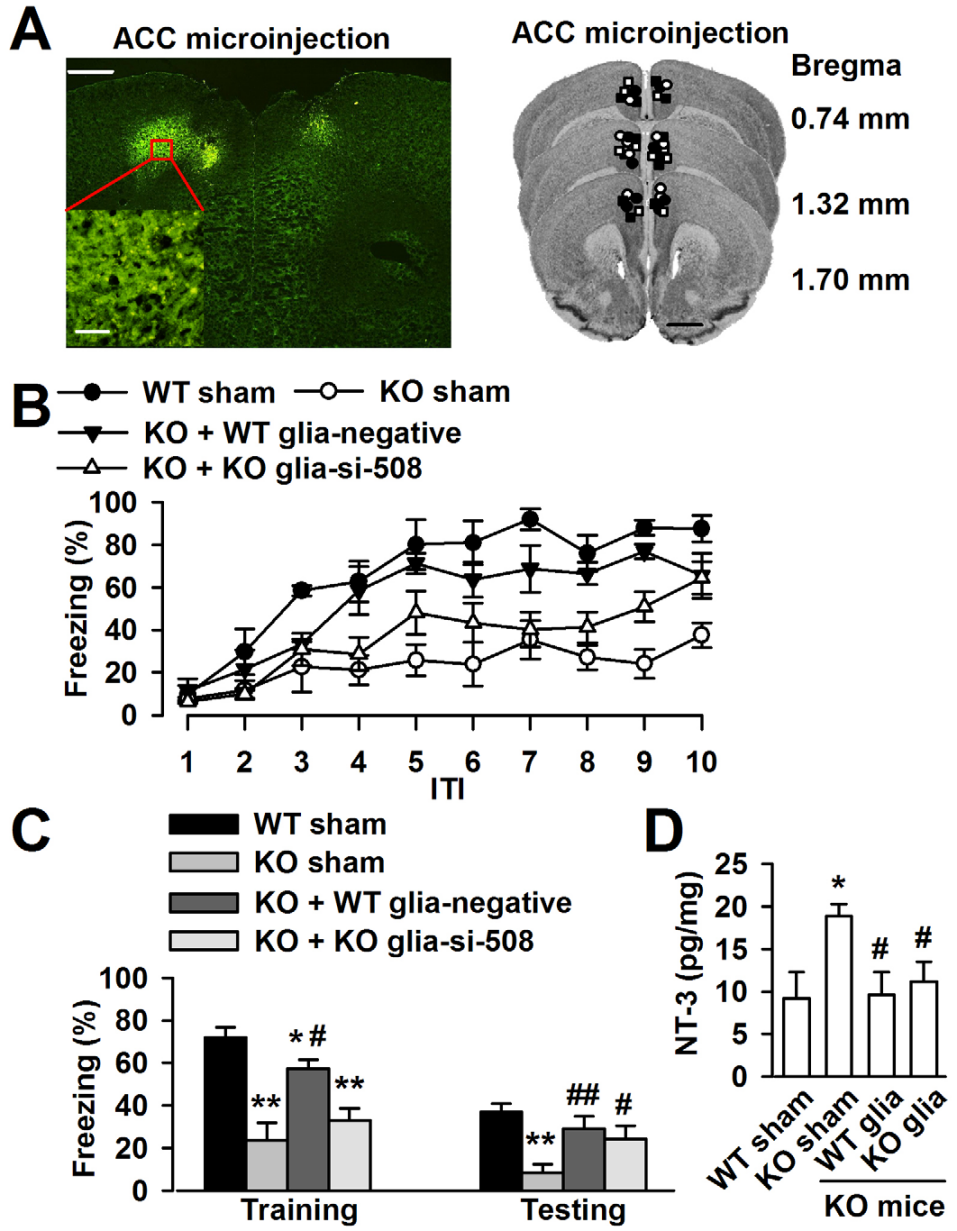
Down-regulation astrocyte-derived NT-3 *in vivo* reversed the trace fear memory of *Fmr1* KO mice. A, ACC section (left upper) showing bilateral GFP-expressing astrocytes 10 days after microinjection. Scale bar, 500 µm; insert: scale bar, 50 µm. Right column showing cannula tip placements in the ACC of WT sham (solid circles), KO sham (open circles), KO mice injected with negative shRNA infected WT astrocytes (solid triangles) or NT-3 shRNA infected KO astrocytes (open triangles). B, ACC microinjection with negative shRNA infected WT astrocytes and si-508 shRNA infected KO astrocytes showed significantly reduced freezing time as compared to KO sham mice during trace fear condition. *n = *6 mice in WT and KO sham group, *n* = 5 in KO mice injected with negative shRNA infected WT astrocytes and si-508 shRNA infected KO astrocytes group. C, Transplantation of negative shRNA infected WT astrocytes and si-508 shRNA infected KO astrocytes in KO mice exhibit reduced average freezing within the intertrial intervals of the training and testing sessions. *n = *6 mice in WT and KO sham group, *n* = 5 in KO mice injected with negative shRNA infected WT astrocytes and si-508 shRNA infected KO astrocytes group. *P<0.05, ** *P*<0.01 compared with the WT sham; ^#^ *P*<0.05, ^##^ *P*<0.01compared with the KO sham. D, Levels of the NT-3 were significantly decreased in the ACC of KO mice after local injection of negative shRNA infected WT astrocytes and si-508 shRNA infected KO astrocytes. *n = *6 mice in each group. *P<0.05 compared with the WT sham; ^#^ *P*<0.05 compared with the KO sham.

To determine whether the cognitive defects of *Fmr1* KO mice may be related to the excessive NT-3 in the brain, we want to rescue the phenotype of *Fmr1* KO mice by ACC local injection of negative shRNA infected WT astrocytes or NT-3 shRNA infected KO astrocytes (Figure 9A). Ten days later, trace fear memory and the levels of NT-3 in the ACC were detected. First, mice were tested in the trace fear conditioning paradigm. Trace fear conditioned learning requires an intact ACC [5], [30]. This paradigm differs from the classic delay paradigm in that the animal must sustain attention during the trace interval to learn the CS–US association [30]. The CS, an 80 dB white noise delivered for 15 s, was delivered 30 s before (trace) the US, a 0.7 mA scrambled foot shock. Mice were presented with 10 CS–trace–US trials with an intertrial interval (ITI) of 210 s. One day after training, mice received 10 CS–ITI trials in a novel chamber to test for trace fear memory. Before training, *Fmr1* KO mice displayed similar baseline freezing compared with WT mice (Figure 9B). WT mice successfully learned the trace fear conditioning after 10 CS–US pairings and showed increased freezing throughout the training session. Freezing was also increased in *Fmr1* KO mice with ACC microinjection of negative shRNA infected WT astrocytes or NT-3 shRNA infected KO astrocytes (Figure 9B). The average freezing during the intertrial intervals of the training session was significantly increased in *Fmr1* KO mice with ACC microinjection of negative shRNA infected WT astrocytes or NT-3 shRNA infected KO astrocytes compared with *Fmr1* KO sham mice (Figure 9C). Similarly, *Fmr1* KO mice with ACC microinjection of negative shRNA infected WT astrocytes or NT-3 shRNA infected KO astrocytes showed increased average freezing within the intertrial intervals of the testing session when compared with *Fmr1* KO sham mice (Figure 9C). Then, we found that levels of NT-3 were significantly decreased in the ACC of KO mice after local injection of negative shRNA infected WT astrocytes and the NT-3 shRNA infected KO astrocytes (Figure 9D). These results suggest that down-regulation of NT-3 by microinjection of WT astrocytes or NT-3 shRNA infected KO astrocytes rescues the impairment at acquiring trace fear memory during training as well as in the expression of trace fear memory during testing on the following day.

## Discussion

Emerging evidence strongly suggests that FMRP expressed in astrocytes may play an important role in neuronal development. The current study focuses on the abnormal neuronal dendritic development induced by *Fmr1* null astrocytes and yields five novel findings: (i) excessive astrocyte-derived NT-3 is found in the *Fmr1* KO astrocyte culture medium and *Fmr1* KO cerebral cortex; (ii) *NT-3* mRNA interacts with FMRP, a protein which is thought to negatively regulate translation; (iii) excessive NT-3 reduces neural dendritic development and synaptic protein levels; (iv) knockdown of NT-3 levels in KO astrocytes restores neuronal dendritic growth; (v) down-regulation astrocyte-derived NT-3 in the prefrontal cortex rescued the deficit of trace fear memory in the KO mice. This study implies a potential therapeutic target of astrocytes for FXS.

### Astrocytes and the altered neurobiology of FXS

FMRP is expressed in astrocytes, oligodendrocytes, and microglia in addition toneurons [19], [31]. The role of astrocytes in the altered neurobiology of FXS has been first demonstrated by Jacobs and Doering, showing that *Fmr1* KO astrocytes have profound effects on dendrites such as reduced length of dendrites and arbor area [20]. Using a coculture design, they found that hippocampal neurons exhibited abnormal dendritic morphology and a decreased number of presynaptic and postsynaptic protein aggregates when they were grown on astrocytes from a Fragile X mouse. Moreover, normal astrocytes could prevent the development of abnormal dendrite morphology and preclude the reduction of presynaptic and postsynaptic protein clusters in neurons from a Fragile X mouse. These experiments established a role for astrocytes in the altered neurobiology of FXS [20]. The further study shows that hippocampal neurons grown on Fragile X astrocytes exhibited delayed growth characteristics and abnormal morphological features in dendrites and synapses [32]. The present paper confirms these results and extends them to show that NT-3 is involved. In this study, astrocytic conditioned medium culture protocols were applied to demonstrate that KO astrocytes affect neuronal growth through altered secretion of soluble factors.

### Neurotrophic factors and development of neurons

Mutant products in astrocytes and microglia can damage neighboring neurons in some neurodegenerative disorders either by releasing toxic components or by mutant-mediated reduction in neuronal support functions [33]. NGF has been reported to decrease the survival of cultured cerebellar granule cells [34] or increase the number of cell deaths in the developing isthmo-optic nucleus [35]. Moreover, high levels of NT-3 are expressed in autism [36] and bipolar disorders [37]. In addition to neurons, astrocytes can represent an important local cellular source of neurotrophins, including NGF, BDNF, GDNF, CNTF, and NT-3 [38], [39]. To investigate the causes for abnormalities of neuronal morphology, we hypothesized the absence or down-regulation of certain neurotrophic factors in KO ACM. The neurotrophic factors are a group of proteins that promote the survival and growth of neurons in the vertebral nervous system. However, in contrast with our expectation, we found higher levels of NT-3 in KO ACM and that excessive NT-3 was toxic to neuronal growth. It has been reported that exogenous NT-3 increases the total number of neuritis of neural plate explants but also the level of apoptosis in early neural development, and that blockade of NT-3 using an antibody reverses these effects [40]. In the present culture system, neuron-derived neurotrophic factors were undetectable. Thus, the significant finding of this study is the neurotoxicity observed only with excessive astrocyte-derived NT-3 from KO mice, which is traditionally considered a critical regulatory factor in cell proliferation, differentiation, and elimination of excess neurons produced during the course of normal brain development [41].

In addition, the overexpression of NT-3 was also detected in the cerebral cortex of *Fmr1* KO mice and the levels of NT-3 in the brain tissue were lower than that in the culture medium. The diminution of the NT-3 difference *in vivo* and *in vitro* could be explained by the involvement of neurons and the state of the astrocytes. NT-3 is produced not only by astrocytes but also by neurons [38], [39]. The levels of NT-3 in the brain can be influenced by hormones [42] and other members of the neurotrophin family [43], [44]. The cultured astrocytes produce NT-3 [39]; however, in the brain (*in vivo*), the reactive gliosis occurs in response to damage to the CNS and the content of neurotrophic factor in reactive gliosis is significantly higher relative to normal astrocytes [38], [45]. Actually, the source of the NT-3 in the cerebral cortex samples in the present study cannot be distinguished.

Two types of receptors that vary in terms of ligand binding specificity mediate the effect of neurotrophins. The low-affinity neurotrophin receptor p75 is capable of binding with all neurotrophins with equivalent affinity, whereas tyrosine kinase (Trk) family members exhibit ligand selectivity. The TrkC receptor is unique as it binds only with NT-3 and not with any other related ligands [46]. NT-3 at high concentration has also been reported to block the survival normally seen with BDNF [47]. In this study, we found that the neuronal damage by NT-3 was dose dependent, and the tremendous neurotoxicity was revealed by 300 pg/ml of NT-3. The differences in the effects of trophic factors between neuronal populations may be due to the different properties inherent in the cells studied, i.e. varying expression levels of different Trk p75 receptors and different responses to environmental changes and lesions [35]. However, physiological levels of NT-3 are also necessary to dendritic development. By applying NT-3 neutralizing antibody, we found a classic bell-shaped curve of the dendritic growth in response to the dose of NT-3 antibody. With the increase of dose, NT-3 antibody gradually ameliorated dendritic growth. The peak dose was 2 µg/ml, higher doses of NT-3 antibody (20 and 40 µg/ml) exerted less effects to the dendritic growth. This suggests that physiological levels of NT-3 are necessary; however, excessive levels are harmful to neuronal dendritic development.

### Translational regulation of FMRP

In this study, we used two biological methods to verify the function of NT-3 in neuronal development, i.e. the transfection of FMRP constructs into cultured *Fmr1* KO astrocytes and knockdown of NT-3 using shRNAs. Both of them could partially rescue the neuronal dendritic morphology disorder. Further RNA-binding protein immunoprecipitation showed that *NT-3* mRNA in FMRP immunoprecipitates in WT astrocytes. As we know, FMRP is associated with polyribosomes and involved in the translational efficiency and/or trafficking of certain mRNAs [7]. These results suggest that excessive NT-3 might be caused by a loss of translation repression of *NT-3* mRNA due to the lack of the FMRP in *Fmr1* KO astrocytes.

### Clinical significance

*In vitro* neutralization against NT-3 provided direct evidence that excessive NT-3 contributes to abnormal dendritic morphology. However, the classic bell-shaped curve depicted dendritic growth in response to NT-3 antibody, suggesting that physiological levels of NT-3 are also necessary todendritic development. Further, *in vivo* ACC microinjection of WT astrocytes or NT-3 shRNA transfected KO astrocytes induced a significant reduction of the NT-3 level in the ACC and rescued the impairment of trace fear memory in the KO mice. Thus, the data presented here provide a possible explanation for the role of astrocytes in the abnormal neuronal dendritic development of FXS and may provide insights into the cellular mechanisms underlying Fragile X syndrome.

## Materials and Methods

### Animals

*Fmr1* KO (FVB.129P2-Fmr1tm1Cgr/J; stock #4624) and control [FVB.129P2-Pde6b+ Tyrc-ch/AntJ; stock #4828; hereafter referred to as wild type (WT)] strain mice were obtained from The Jackson Laboratory. Mice were housed under a 12 h light/dark cycle with food and water provided *ad libitum*. All animal protocols used were approved by the Animal Care and Use Committee of the Fourth Military Medical University.

### Primary neural cultures

The primary cultures of the cortical neurons were isolated from 15-day-old embryos (E15) of *Fmr1* WT and KO mice [14]. Twenty-four hours after plating with Dulbecco's Modified Eagle Medium (DMEM) containing 20% fetal bovine serum (FBS) (Invitrogen, Carlsbad, USA), the medium was completely changed into a neurobasal medium with 2% B27 supplement (Invitrogen). Half of the medium was changed every three days, and the neurons were used in the experiments at 7 days *in vitro* (DIV). The primary astrocytes were isolated from either WT or *Fmr1* KO one- (P1) to two-day-old (P2) pups using the differential adhesion method [48]. The dissociated cells were placed in a 25 cm^2^ tissue culture flask in DMEM containing 10% FBS and 2 mM of glutamine. When confluent (after 6 to 7 days), the flasks were sealed and shaken at 250 rpm at 37°C for 16 h. Over 95% of the adherent cells were astrocytes as demonstrated by the anti-glial fibrillary acidic protein (anti-GFAP) immunostaining.

### The coculture of cortical neuron and astrocytes

The coculture of the neurons with astrocytes was performed as described previously [22]. The neurons were plated onto cover slips containing paraffin dots in neuronal plating medium. The cover slips were taken out after 4 h when the neurons adhered and then placed in 24-well culture plates containing a monolayer of astrocytes (50% to 60% confluence) in the NB/B27 neuronal maintenance medium after inversion.

### Preparation of ACM and culturing neurons in ACM

The astrocytes were digested with 0.25% trypsin (Invitrogen) plus 0.02% ethylenediaminetetraacetic acid (EDTA) and then passaged to a 25 cm^2^ tissue culture flask. The cells were confluent after three days. The cultures were washed extensively with Hank's balanced salt solution (HBSS) (Invitrogen), and the medium was replaced with NB/B27 culture medium to generate the ACM. After which, the ACM were collected at 7 DIV as previously described [22]. To culture the neurons in ACM, we isolated the cortical neurons and plated them on poly-L-lysine-coated plates containing the neuronal plating medium. The medium was kept for 4 h and then replaced by a 1∶1 mixture of ACM and neuronal maintenance medium to ensure that the neurons have adhered to the plate surfaces. Half of the medium was changed every three days throughout the cultures.

### Immunofluorescence staining and quantification

The neurons were fixed in 4% paraformaldehyde for 20 min at room temperature after being cultured for seven days. After blocking for 30 min (5% BSA and 0.1% TritonX-100), the cells were stained with anti-MAP2 (1∶1000, Millipore, Billerica, USA) overnight at 4°C followed by secondary Cy3-conjugated anti-rabbit antibody (1∶200, Boster Bio-Technology, Wuhan, China). The astrocytes were similarly fixed and stained with anti-GFAP (1∶2000, Millipore) and secondary Alexa488-conjugated anti-mouse antibody (1∶800, Invitrogen). The neurons and astrocytes were stained with Hoechst33258 (10 µg/ml, Beyotime Institute Biotechnology, Shanghai, China) to observe their nuclei. The slides were observed using a confocal laser microscope (FV1000, Olympus, Tokyo), and the images were captured using Fluoview 1000 (Olympus). For quantification of cell morphology, photomicrographs were taken randomly from each culture condition. The area of astrocytes and total dendritic length of isolated neurons were measured by ImageJ software [20], [22]. The photography and analysis of immunoreactivity were performed in an investigator-blinded manner in three independent experiments. Each experiment used three or more cover slips to obtain the sample of isolated neurons for analysis.

### Western blot analysis

The cells were lysated in a RIPA lysis buffer (Beyotime Institute Biotechnology) and then briefly sonicated. Equivalent amounts of protein were resolved using 10% SDS-PAGE gel and transferred to the nitrocellulose membrane. After incubation with antibodies, the proteins were observed using enhanced chemiluminescence (ECL, GE Healthcare Pharmacia, Uppsala, Sweden). The following primary antibodies were used: anti-FMRP (1∶1000; Millipore), anti-GFAP (1∶1000; Millipore), anti-MAP2 (1∶1000; Millipore), anti-PSD95 (1∶2000; Abcam, Cambridge, UK), anti-GluR1 (1∶300; Abcam), anti-NT-3 (1∶200; Santa Cruz, CA, USA), anti-TrkC (1∶200; Santa Cruz), and anti-β-actin (1∶10000; Sigma, St. Louis, MO). The secondary antibodies were horseradish peroxidase conjugated goat antibody to rabbit or mouse immunoglobulin (1∶10000; Boster Bio-Technology). The densitometric analysis of Western blots was conducted using a ChemiDoc XRS (Bio-Rad, Hercules, CA) and quantified using Quantity One version 4.1.0 (Bio-Rad).

### ABC ELISA for neurotrophic factors

The ACM samples were collected after brief centrifugation. The cerebral cortex tissues were normalized via Bicinchoninic Acid (BCA) Protein Assay to generate homogenates. The activation was evaluated by measuring the nerve growth factor (NGF), neurorophin-3 (NT-3), brain-derived neurotrophic factor (BDNF), glial cell-derived neurotrophic factor (GDNF), and ciliary neurotrophic factor (CNTF) in the medium using enzyme-linked immunosorbent assay (ELISA) kits according to the manufacturer's instruction (Westang, Shanghai, China). The concentration of these neurotrophic factors was detected using Denley Dragon Wellscan MK 3 and quantified using Ascent software for Multiskan.

### Construction and transfection of FMRP expression vector

Full-length mouse *Fmr1* was produced by reverse transcription-polymerase chain reaction (RT-PCR) using the following primers: 5′-CGGAATTC(EcoRI) GACGAGAAGATGGAGGAG-3′ and 5′-CCCTCGAG(XhoI)TACGGAAATGGTAGAGGA-3′. The purified PCR product was cloned into the pIRES2-EGFP vector with the corresponding restriction enzyme sites (EcoRI/XhoI). The recombinant DNA was confirmed by sequencing, and the expression of correctly sized proteins was confirmed via Western blot using an anti-FMRP antibody. The FMRP expression vector or an empty vector (control) were transfected using Lipofectamine LTX and PLUS Reagents (Invitrogen) according to the manufacturer's instructions.

### Real-time PCR analysis of *NT-3* mRNA

The WT and KO astrocytes were collected, and the total RNA was isolated from each sample using a Trizol reagent (Invitrogen) according to the manufacturer's instructions. The total RNA (2 µg) was reverse transcribed using reverse transcriptase (TaKaRa Biotechnology). The first strand cDNA was used as the template for real-time quantitative PCR analysis. The primers used were as follows: 5′-CATGTCGACGTCCCTGGAAATAG-3′ (forward) and 5′-GGATGCCACGGAGATAAGCAA-3′ (reverse) for *NT-3* and 5′-TGTGTCCGTCGTGGATCTGA-3′ (forward) and 5′-TTGCTGTTGAAGTCGCAGGAG-3′ (reverse) for the internal quantitative control *GAPDH*. The mRNAs were detected using SYBR Green PCR Master Mix (TaKaRa Biotechnology) and an ABI PRISM 7500 Sequence Detection System (Applied Biosystems, UK) using the comparative threshold cycle method for relative quantification. The thermal cycling conditions were as follows: 95°C for 30 s, 40 cycles of 95°C for 5 s, and 60°C for 34 s.

### RNA–binding protein immunoprecipitation (RIP) and RT–PCR

The RIP analysis was performed using the Magna RIP Kit (Millipore Bioscience Research Reagents). The primary astrocytes were dispersed in an appropriate volume of complete RIP lysis buffer. The magnetic beads for immunoprecipitation were prepared and subsequently incubated for 30 min at room temperature with 5 µg anti-FMRP antibody (Millipore Bioscience Research Reagents). The immunoprecipitation of the RIP lysate and beads-antibody complex was performed at 4°C overnight. After the protein degradation with proteinase K at 55°C for 30 min, the RNA was extracted using phenol/chloroform/isoamyl alcohol and precipitated with ethanol. RT-PCR then was performed using gDNA Eraser (TaKaRa Biotechnology). The following primers were used: *NT-3*, 5′-GGGGTGGGCGAGACTGAATG-3′ (forward) and 5′-TCCCTGGGTGCCTCTGCTTT-3′ (reverse); *BDNF*, 5′-TGCCAGTTGCTTTGTCTTCT-3′ (forward) and 5′-AGTGTCAGCCAGTGATGTCG-3′ (reverse); *NGF*, 5′-TGAAGCCCACTGGACTAAA-3′ (forward) and 5′- ACCTCCTTGCCCTTGATG-3′ (reverse); the positive control *MAP1B*, 5′-GGCAAGATGGGGTATAGAGA-3′ (forward) and 5′- CCCACCTGCTTTGGTATTTG-3′ (reverse); and the negative control *GAPDH*, 5′-TTAGCCCCCCTGGCCAAGG-3′ (forward) and 5′-CTTACTCCTTGGAGGCCATG-3′ (reverse). The PCR products were separated and visualized on an agarose gel containing 5 g/l ethidium bromide.

### Neurotrophin neutralization

In an attempt to block the neurotoxicity of excessive astrocyte-derived NT-3, neutralizing antibody to NT-3 (anti- human NT-3 pAb, catalog No. G1651; Promega, Madison, WI, USA) was added to the KO ACM cultured wells on the day of plating and for the entire time in cultures (7 days). Normal rabbit IgG (Santa Cruz) was administered as a negative control. The antibodies and normal rabbit IgG were resuspended in sterile PBS. The neutralizing specificity has been demonstrated previously [49], [50]. In the present culture system, this neutralizing antibody was used at a final concentration of 0.02, 0.2, 2, 20, and 40 µg/ml, respectively.

### Lentiviral constructs and shRNA for NT-3

The NT-3 shRNAs lentiviruses were packaged by Genepharma (Shanghai, China). Three sequences (si-462: GGTCAGAGTTCCAGCCAATGA, si-508: GCAACAGAGACGCTA CAATTC, and si-889: GCAAACCTATGTCCGAGCACT) were designed to reduce NT-3 expression compared with cells infected with lentivirus containing a nonsense control sequence (negative: TTCTCCGAACGTGTCACGT). For the infection process, the astrocytes were plated onto 10 cm cell culture dishes and grown to 30% to 50% confluence before infection. The cells were incubated with lentivirus in serum-free conditions for 24 h at 37°C and then rinsed with PBS. After being incubated in DMEM containing 10% FBS for another 24 h, the cells were treated with NB/B27 and the ACM was collected.

### Cell transplantation and behavioral tests

Before transplantation *in vivo*, the cultured WT and KO astrocytes were infected by negative and si-508 NT-3 lentivirus, respectively. Two days later, the infected astrocytes were carried with green fluorescence, then digested and resuspended at a concentration of 1×10^4^ cells/µl in PBS. The experimental mice were 3∼4 weeks old, and divided into four groups, each group containing 5 mice. Two groups of *Fmr1* KO mice were anesthetized with pentobarbital sodium (1 mg/20 g body weight, intraperitoneally) and cells were stereotaxically microinjected into bilateral anterior cingulate cortex (ACC; 1 mm anterior to the bregma, ±0.3 mm lateral from the midline, and 1.4 mm beneath the surface of the skull) at a rate of 1 µl/min and at a volume of 1 µl/side, resulting in a dose of 1×10^4^ cells per side. The other two groups were WT and KO mice sham operated controls, respectively. The mice were subjected to the same surgery but without cells in PBS. To confirm the localization and vitality of the injected cells in ACC, the brain was prepared from a surgical mouse and perfused with physiological saline, followed by fixative solution of 4% paraformaldehyde. The brain was post-fixed, sucrose-dehydrated and frozen-sliced at 20 µm thickness using a freezing microtome (CM1950, Leica, Germany). A coronal section including the injection's nick was selected and processed for green fluorescence observation.

After ten days of surgery, trace fear conditioning was performed in an isolated shock chamber (Med Associates, St. Albans, VT). The CS used was an 80 dB white noise, delivered for 15 s, and the US was a 0.7 mA scrambled footshock for 0.5 s. Mice were acclimated for 60 s and were presented with 10 CS–trace–US intertrial interval [31] trials (trace, 30 s; ITI, 210 s). One day after training, mice were acclimated for 60 s followed by 10 CS–ITI trials (ITI, 210 s) in a novel chamber to test for trace fear memory [5], [51], [52]. All data were video recorded using FreezeFrame Video-Based Conditioned Fear System and analyzed by Actimetrics Software (Coulbourn Instruments, Wilmette, IL). Average freezing for the baseline and for each ITI during the training and testing sessions was analyzed. Bouts of 1.0 s were used to define freezing.

### Statistical analyses

The data were expressed as mean±SEM. The statistical comparisons were performed via analysis of variance (ANOVA). If the ANOVA was significant, *post hoc* comparisons were conducted using Tukey's test. In all cases, *P*<0.05 was considered statistically significant.

## Supporting Information

Figure S1 High levels of NT-3 protein in KO astrocytes. A, The levels of *NT-3* mRNA were assessed by real time PCR assays. There was no difference between WT and KO astrocytes. Data were from three independent experiments. B, The expression of NT-3 protein levels in WT and KO astrocytes were detected by Western blot. C, Band intensity analysis showed NT-3 protein levels in KO astrocytes were higher than in WT astrocytes. *n = *6 wells from three independent experiments. **P<0.01 compared with the WT astrocytes.
(JPG)

Figure S2 Effects of excessive NGF and BDNF on neuronal development. A, High levels of exogenous NGF and BDNF had no neurotoxicity to neuronal development. Scale bar = 50 µm. B, Quantification of neurons with at least two short (<50 µm) dendrites. C, Quantification of the total dendritic length per cell. B–C: the number of neurons in WT ACM: *n = *242 neurons, KO ACM: *n = *215 neurons, WT ACM + 150 pg/ml NGF: *n = *248 neurons, WT ACM + 300 pg/ml NGF: *n = *235 neurons, WT ACM + 150 pg/ml BDNF: *n = *255 neurons, WT ACM + 300 pg/ml BDNF: *n = *274 neurons. Data were from three independent experiments. **P<0.01 compared with the WT ACM.
(JPG)

Figure S3 Knockdown of NT-3 in *Fmr1* KO astrocytes rescued the synaptic proteins. A, The expressions of MAP2, PSD95, and GluR1 were detected by Western blot. B, Band intensities showed ACM from KO astrocytes infected with si-508 and si-889 shRNAs reversed the decreased levels of MAP2, PSD95, and GluR1 in KO ACM-treated neurons. *n = *6 wells from three independent experiments. *P<0.05, **P<0.01 compared with the KO ACM-treated group.
(JPG)

## References

[1] Feng Y, Zhang F, Lokey LK, Chastain JL, Lakkis L, et al (1995) Translational suppression by trinucleotide repeat expansion at FMR1. Science 268: 731–734. 7732383 10.1126/science.7732383

[2] Pieretti M, Zhang FP, Fu YH, Warren ST, Oostra BA, et al (1991) Absence of expression of the FMR-1 gene in fragile X syndrome. Cell 66: 817–822. 1878973 10.1016/0092-8674(91)90125-i

[3] Bagni C, Greenough WT (2005) From mRNP trafficking to spine dysmorphogenesis: the roots of fragile X syndrome. Nat Rev Neurosci 6: 376–387. 15861180 10.1038/nrn1667

[4] Bear MF, Huber KM, Warren ST (2004) The mGluR theory of fragile X mental retardation. Trends Neurosci 27: 370–377. 15219735 10.1016/j.tins.2004.04.009

[5] Zhao MG, Toyoda H, Ko SW, Ding HK, Wu LJ, et al (2005) Deficits in trace fear memory and long-term potentiation in a mouse model for fragile X syndrome. J Neurosci 25: 7385–7392. 16093389 10.1523/JNEUROSCI.1520-05.2005 PMC6725289

[6] Skinner M, Hooper S, Hatton DD, Roberts J, Mirrett P, et al (2005) Mapping nonverbal IQ in young boys with fragile X syndrome. Am J Med Genet A 132A: 25–32. 15551333 10.1002/ajmg.a.30353

[7] Jin P, Warren ST (2003) New insights into fragile X syndrome: from molecules to neurobehaviors. Trends Biochem Sci 28: 152–158. 12633995 10.1016/S0968-0004(03)00033-1

[8] Bakker CE, de Diego Otero Y, Bontekoe C, Raghoe P, Luteijn T, et al (2000) Immunocytochemical and biochemical characterization of FMRP, FXR1P, and FXR2P in the mouse. Exp Cell Res 258: 162–170. 10912798 10.1006/excr.2000.4932

[9] Devys D, Lutz Y, Rouyer N, Bellocq JP, Mandel JL (1993) The FMR-1 protein is cytoplasmic, most abundant in neurons and appears normal in carriers of a fragile X premutation. Nat Genet 4: 335–340. 8401578 10.1038/ng0893-335

[10] Miyashiro KY, Beckel-Mitchener A, Purk TP, Becker KG, Barret T, et al (2003) RNA cargoes associating with FMRP reveal deficits in cellular functioning in Fmr1 null mice. Neuron 37: 417–431. 12575950 10.1016/s0896-6273(03)00034-5

[11] Todd PK, Mack KJ, Malter JS (2003) The fragile X mental retardation protein is required for type-I metabotropic glutamate receptor-dependent translation of PSD-95. Proc Natl Acad Sci U S A 100: 14374–14378. 14614133 10.1073/pnas.2336265100 PMC283599

[12] Consortium TD-BFX (1994) Fmr1 knockout mice: a model to study fragile X mental retardation. Cell 78: 23–33. 8033209

[13] Frankland PW, Wang Y, Rosner B, Shimizu T, Balleine BW, et al (2004) Sensorimotor gating abnormalities in young males with fragile X syndrome and Fmr1-knockout mice. Mol Psychiatry 9: 417–425. 14981523 10.1038/sj.mp.4001432

[14] Wang H, Wu LJ, Kim SS, Lee FJ, Gong B, et al (2008) FMRP acts as a key messenger for dopamine modulation in the forebrain. Neuron 59: 634–647. 18760699 10.1016/j.neuron.2008.06.027

[15] Haber M, Zhou L, Murai KK (2006) Cooperative astrocyte and dendritic spine dynamics at hippocampal excitatory synapses. J Neurosci 26: 8881–8891. 16943543 10.1523/JNEUROSCI.1302-06.2006 PMC6675342

[16] Nishida H, Okabe S (2007) Direct astrocytic contacts regulate local maturation of dendritic spines. J Neurosci 27: 331–340. 17215394 10.1523/JNEUROSCI.4466-06.2007 PMC6672072

[17] Pfrieger FW, Barres BA (1997) Synaptic efficacy enhanced by glial cells in vitro. Science 277: 1684–1687. 9287225 10.1126/science.277.5332.1684

[18] Ullian EM, Sapperstein SK, Christopherson KS, Barres BA (2001) Control of synapse number by glia. Science 291: 657–661. 11158678 10.1126/science.291.5504.657

[19] Pacey LK, Doering LC (2007) Developmental expression of FMRP in the astrocyte lineage: implications for fragile X syndrome. Glia 55: 1601–1609. 17823967 10.1002/glia.20573

[20] Jacobs S, Doering LC (2010) Astrocytes prevent abnormal neuronal development in the fragile x mouse. J Neurosci 30: 4508–4514. 20335488 10.1523/JNEUROSCI.5027-09.2010 PMC6634485

[21] Beckel-Mitchener A, Greenough WT (2004) Correlates across the structural, functional, and molecular phenotypes of fragile X syndrome. Ment Retard Dev Disabil Res Rev 10: 53–59. 14994289 10.1002/mrdd.20009

[22] Ballas N, Lioy DT, Grunseich C, Mandel G (2009) Non-cell autonomous influence of MeCP2-deficient glia on neuronal dendritic morphology. Nat Neurosci 12: 311–317. 19234456 10.1038/nn.2275 PMC3134296

[23] Ip NY, Boulton TG, Li Y, Verdi JM, Birren SJ, et al (1994) CNTF, FGF, and NGF collaborate to drive the terminal differentiation of MAH cells into postmitotic neurons. Neuron 13: 443–455. 8060620 10.1016/0896-6273(94)90359-x

[24] Ventimiglia R, Mather PE, Jones BE, Lindsay RM (1995) The neurotrophins BDNF, NT-3 and NT-4/5 promote survival and morphological and biochemical differentiation of striatal neurons in vitro. Eur J Neurosci 7: 213–222. 7757258 10.1111/j.1460-9568.1995.tb01057.x

[25] Lohof AM, Ip NY, Poo MM (1993) Potentiation of developing neuromuscular synapses by the neurotrophins NT-3 and BDNF. Nature 363: 350–353. 8497318 10.1038/363350a0

[26] Wang H, Ku L, Osterhout DJ, Li W, Ahmadian A, et al (2004) Developmentally-programmed FMRP expression in oligodendrocytes: a potential role of FMRP in regulating translation in oligodendroglia progenitors. Hum Mol Genet 13: 79–89. 14613971 10.1093/hmg/ddh009

[27] Zalfa F, Giorgi M, Primerano B, Moro A, Di Penta A, et al (2003) The fragile X syndrome protein FMRP associates with BC1 RNA and regulates the translation of specific mRNAs at synapses. Cell 112: 317–327. 12581522 10.1016/s0092-8674(03)00079-5

[28] Lu R, Wang H, Liang Z, Ku L, O'Donnell W T, et al (2004) The fragile X protein controls microtubule-associated protein 1B translation and microtubule stability in brain neuron development. Proc Natl Acad Sci U S A 101: 15201–15206. 15475576 10.1073/pnas.0404995101 PMC524058

[29] Zalfa F, Eleuteri B, Dickson KS, Mercaldo V, De Rubeis S, et al (2007) A new function for the fragile X mental retardation protein in regulation of PSD-95 mRNA stability. Nat Neurosci 10: 578–587. 17417632 10.1038/nn1893 PMC2804293

[30] Han CJ, O'Tuathaigh CM, van Trigt L, Quinn JJ, Fanselow MS, et al (2003) Trace but not delay fear conditioning requires attention and the anterior cingulate cortex. Proc Natl Acad Sci U S A 100: 13087–13092. 14555761 10.1073/pnas.2132313100 PMC240749

[31] Yuskaitis CJ, Beurel E, Jope RS (2010) Evidence of reactive astrocytes but not peripheral immune system activation in a mouse model of Fragile X syndrome. Biochim Biophys Acta 1802: 1006–1012. 20600866 10.1016/j.bbadis.2010.06.015 PMC2942952

[32] Jacobs S, Nathwani M, Doering LC (2010) Fragile X astrocytes induce developmental delays in dendrite maturation and synaptic protein expression. BMC Neurosci 11: 132. 20955577 10.1186/1471-2202-11-132 PMC2970604

[33] Lobsiger CS, Cleveland DW (2007) Glial cells as intrinsic components of non-cell-autonomous neurodegenerative disease. Nat Neurosci 10: 1355–1360. 17965655 10.1038/nn1988 PMC3110080

[34] Segal RA, Takahashi H, McKay RD (1992) Changes in neurotrophin responsiveness during the development of cerebellar granule neurons. Neuron 9: 1041–1052. 1463606 10.1016/0896-6273(92)90064-k

[35] von Bartheld CS, Kinoshita Y, Prevette D, Yin QW, Oppenheim RW, et al (1994) Positive and negative effects of neurotrophins on the isthmo-optic nucleus in chick embryos. Neuron 12: 639–654. 8155324 10.1016/0896-6273(94)90219-4

[36] Sajdel-Sulkowska EM, Xu M, Koibuchi N (2009) Increase in cerebellar neurotrophin-3 and oxidative stress markers in autism. Cerebellum 8: 366–372. 19357934 10.1007/s12311-009-0105-9

[37] Fernandes BS, Gama CS, Walz JC, Cereser KM, Fries GR, et al (2010) Increased neurotrophin-3 in drug-free subjects with bipolar disorder during manic and depressive episodes. J Psychiatr Res 44: 561–565. 20060128 10.1016/j.jpsychires.2009.11.020

[38] Condorelli DF, Dell'Albani P, Mudo G, Timmusk T, Belluardo N (1994) Expression of neurotrophins and their receptors in primary astroglial cultures: induction by cyclic AMP-elevating agents. J Neurochem 63: 509–516. 7518499 10.1046/j.1471-4159.1994.63020509.x

[39] Rudge JS, Alderson RF, Pasnikowski E, McClain J, Ip NY, et al (1992) Expression of Ciliary Neurotrophic Factor and the Neurotrophins-Nerve Growth Factor, Brain-Derived Neurotrophic Factor and Neurotrophin 3-in Cultured Rat Hippocampal Astrocytes. Eur J Neurosci 4: 459–471. 12106332 10.1111/j.1460-9568.1992.tb00896.x

[40] Li R, Bernd P (1999) Neurotrophin-3 increases neurite outgrowth and apoptosis in explants of the chicken neural plate. Dev Neurosci 21: 12–21. 10077697 10.1159/000017361

[41] Lu B, Pang PT, Woo NH (2005) The yin and yang of neurotrophin action. Nat Rev Neurosci 6: 603–614. 16062169 10.1038/nrn1726

[42] Lindholm D, Castren E, Tsoulfas P, Kolbeck R, Berzaghi Mda P, et al (1993) Neurotrophin-3 induced by tri-iodothyronine in cerebellar granule cells promotes Purkinje cell differentiation. J Cell Biol 122: 443–450. 8320266 10.1083/jcb.122.2.443 PMC2119654

[43] Lindholm D, da Penha Berzaghi M, Cooper J, Thoenen H, Castren E (1994) Brain-derived neurotrophic factor and neurotrophin-4 increase neurotrophin-3 expression in the rat hippocampus. Int J Dev Neurosci 12: 745–751. 7747601 10.1016/0736-5748(94)90054-x

[44] Patz S, Wahle P (2004) Neurotrophins induce short-term and long-term changes of cortical neurotrophin expression. Eur J Neurosci 20: 701–708. 15255980 10.1111/j.1460-9568.2004.03519.x

[45] Wu VW, Nishiyama N, Schwartz JP (1998) A culture model of reactive astrocytes: increased nerve growth factor synthesis and reexpression of cytokine responsiveness. J Neurochem 71: 749–756. 9681466 10.1046/j.1471-4159.1998.71020749.x

[46] Raffioni S, Bradshaw RA, Buxser SE (1993) The receptors for nerve growth factor and other neurotrophins. Annu Rev Biochem 62: 823–850. 8352602 10.1146/annurev.bi.62.070193.004135

[47] Dechant G, Biffo S, Okazawa H, Kolbeck R, Pottgiesser J, et al (1993) Expression and binding characteristics of the BDNF receptor chick trkB. Development 119: 545–558. 8287802 10.1242/dev.119.2.545

[48] McCarthy KD, de Vellis J (1980) Preparation of separate astroglial and oligodendroglial cell cultures from rat cerebral tissue. J Cell Biol 85: 890–902. 6248568 10.1083/jcb.85.3.890 PMC2111442

[49] Rana OR, Schauerte P, Hommes D, Schwinger RH, Schroder JW, et al (2010) Mechanical stretch induces nerve sprouting in rat sympathetic neurocytes. Auton Neurosci 155: 25–32. 20122881 10.1016/j.autneu.2010.01.003

[50] Helke CJ, Verdier-Pinard D (2000) Neurotrophins alter the numbers of neurotransmitter-ir mature vagal/glossopharyngeal visceral afferent neurons in vitro. Brain Res 884: 206–212. 11082504 10.1016/s0006-8993(00)02988-7

[51] Huerta PT, Sun LD, Wilson MA, Tonegawa S (2000) Formation of temporal memory requires NMDA receptors within CA1 pyramidal neurons. Neuron 25: 473–480. 10719900 10.1016/s0896-6273(00)80909-5

[52] Zhao MG, Ko SW, Wu LJ, Toyoda H, Xu H, et al (2006) Enhanced presynaptic neurotransmitter release in the anterior cingulate cortex of mice with chronic pain. J Neurosci 26: 8923–8930. 16943548 10.1523/JNEUROSCI.2103-06.2006 PMC6675332

