# Supplementary figures and images for: Excessive Astrocyte-Derived Neurotrophin-3 Contributes to the Abnormal Neuronal Dendritic Development in a Mouse Model of Fragile X Syndrome
Source: PLoS Genet. 2012 Dec 27;8(12):e1003172. doi: 10.1371/journal.pgen.1003172 (PMC3531466; doi:10.1371/journal.pgen.1003172)

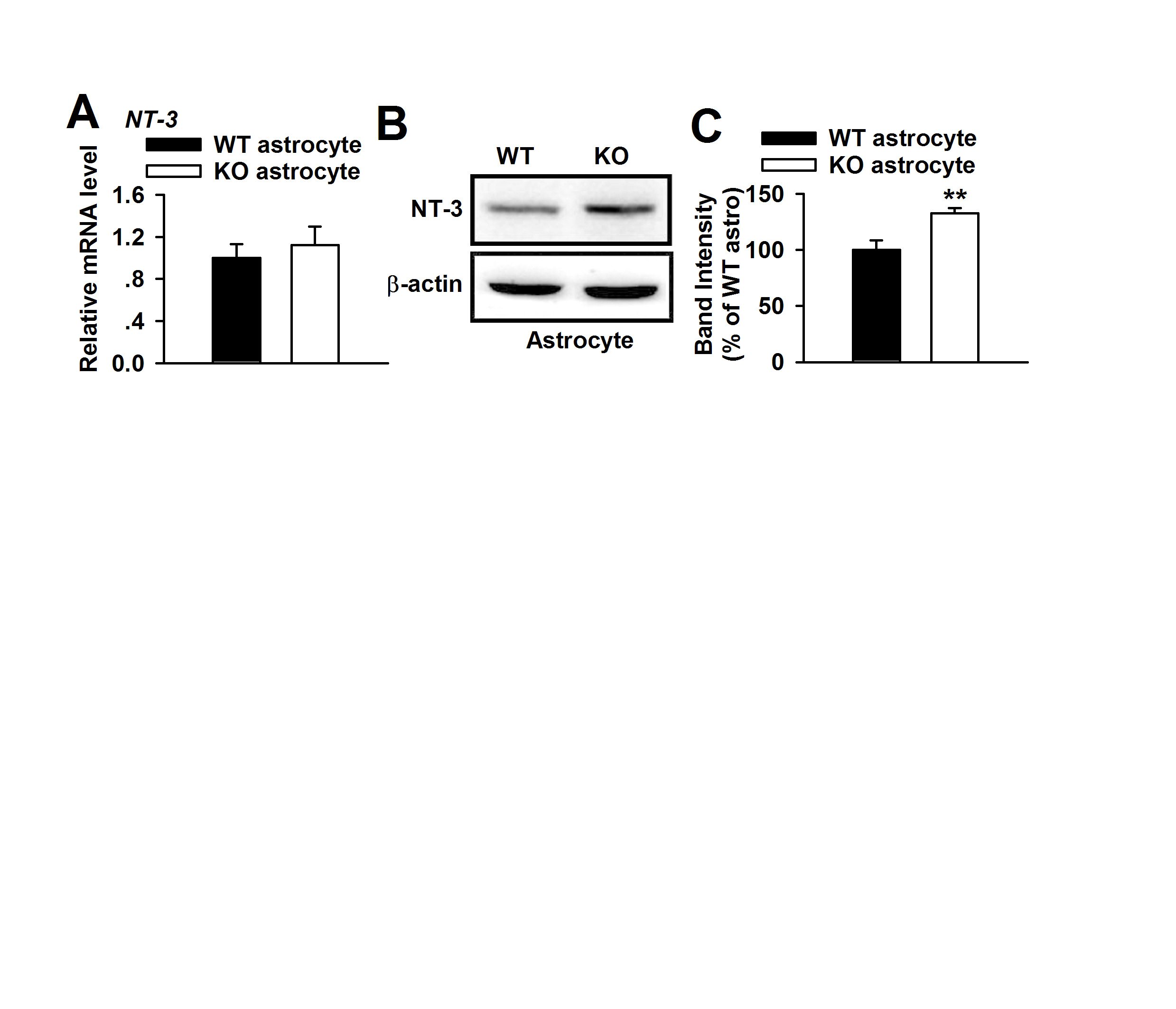

Supplement: Figure S1 — High levels of NT-3 protein in KO astrocytes. A, The levels of NT-3 mRNA were assessed by real time PCR assays. There was no difference between WT and KO astrocytes. Data were from three independent experiments. B, The expression of NT-3 protein levels in WT and KO astrocytes were detected by Western blot. C, Band intensity analysis showed NT-3 protein levels in KO astrocytes were higher than in WT astrocytes. n = 6 wells from three independent experiments. **P<0.01 compared with the WT astrocytes. (JPG) [file pgen.1003172.s001.jpg]

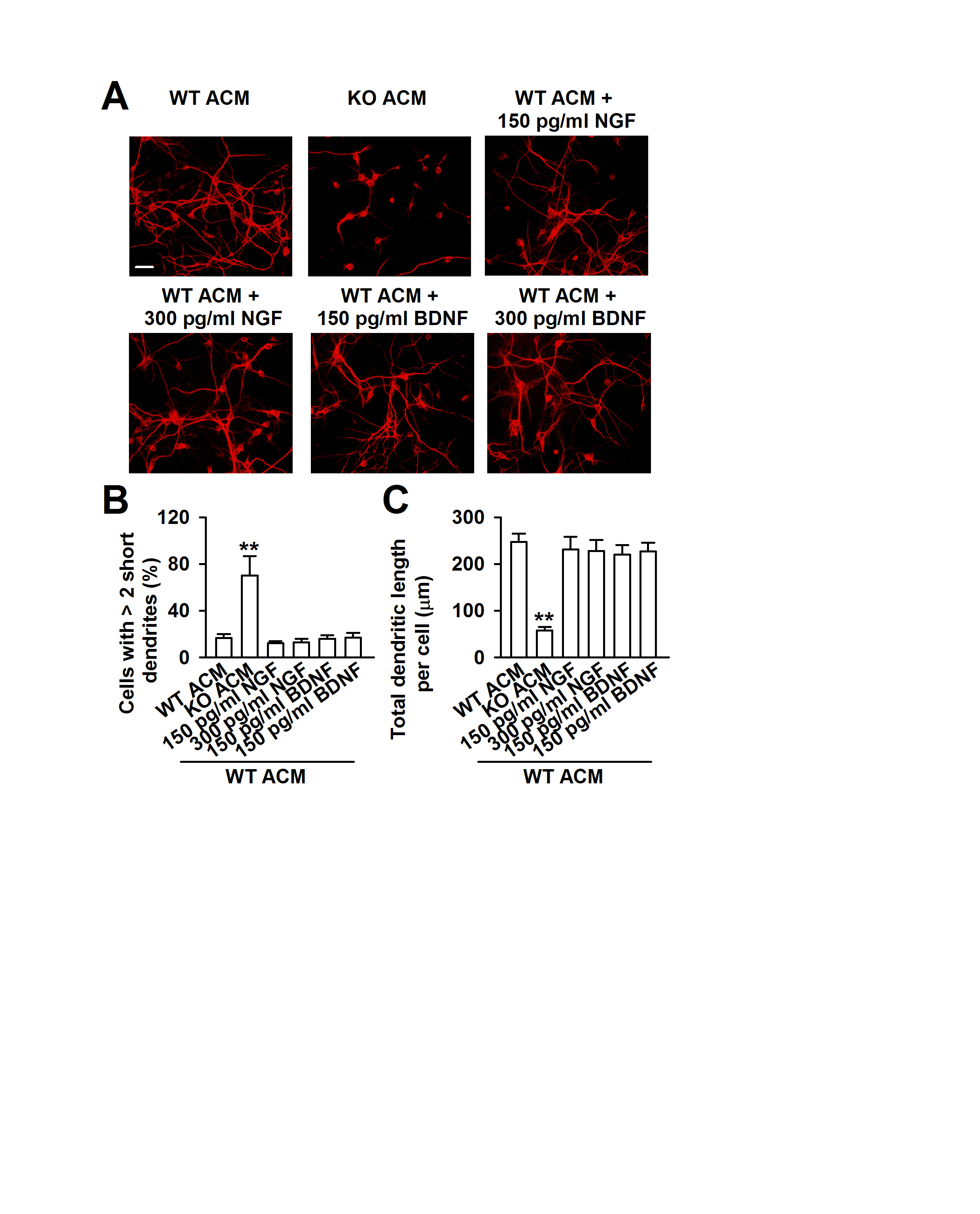

Supplement: Figure S2 — Effects of excessive NGF and BDNF on neuronal development. A, High levels of exogenous NGF and BDNF had no neurotoxicity to neuronal development. Scale bar = 50 µm. B, Quantification of neurons with at least two short (<50 µm) dendrites. C, Quantification of the total dendritic length per cell. B–C: the number of neurons in WT ACM: n = 242 neurons, KO ACM: n = 215 neurons, WT ACM + 150 pg/ml NGF: n = 248 neurons, WT ACM + 300 pg/ml NGF: n = 235 neurons, WT ACM + 150 pg/ml BDNF: n = 255 neurons, WT ACM + 300 pg/ml BDNF: n = 274 neurons. Data were from three independent experiments. **P<0.01 compared with the WT ACM. (JPG) [file pgen.1003172.s002.jpg]

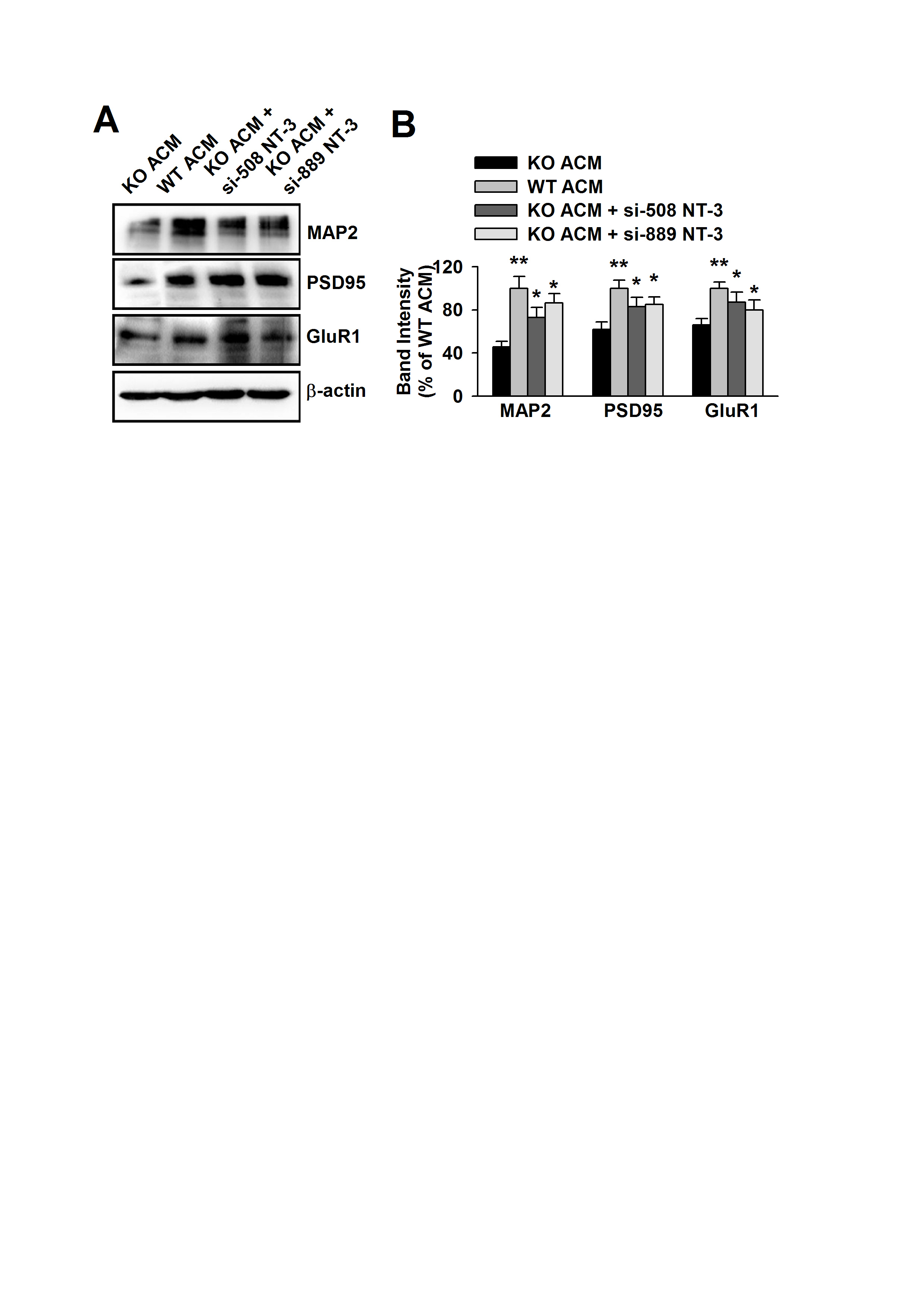

Supplement: Figure S3 — Knockdown of NT-3 in Fmr1 KO astrocytes rescued the synaptic proteins. A, The expressions of MAP2, PSD95, and GluR1 were detected by Western blot. B, Band intensities showed ACM from KO astrocytes infected with si-508 and si-889 shRNAs reversed the decreased levels of MAP2, PSD95, and GluR1 in KO ACM-treated neurons. n = 6 wells from three independent experiments. *P<0.05, **P<0.01 compared with the KO ACM-treated group. (JPG) [file pgen.1003172.s003.jpg]
